# Hospital Dissemination of *tst-1*-Positive Clonal Complex 5 (CC5) Methicillin-Resistant *Staphylococcus aureus*

**DOI:** 10.3389/fcimb.2017.00101

**Published:** 2017-03-31

**Authors:** Min Wang, Yi Zheng, Jose. R. Mediavilla, Liang Chen, Barry. N. Kreiswirth, Yajun Song, Ruifu Yang, Hong Du

**Affiliations:** ^1^State Key Laboratory of Pathogen and Biosecurity, Beijing Institute of Microbiology and EpidemiologyBeijing, China; ^2^Clinical Laboratory, The Second Affiliated Hospital of Soochow UniversitySuzhou, China; ^3^New Jersey Medical School, Public Health Research Institute Tuberculosis Center, Rutgers UniversityNewark, NJ, USA

**Keywords:** MRSA, *spa* typing, SCC*mec* typing, *tst*, CC5, in-hospital 30-day mortality, multivariable analysis

## Abstract

Methicillin-resistant *Staphylococcus aureus* (MRSA), is one of the most prevalent clinical pathogens isolated from hospital settings, and has increasingly identified in community settings. In China, the SCC*mec*III-ST239 strains are disseminated in different geographic regions, accounting for >75% of all MRSA isolates in some national studies. Here we characterized 150 non-duplicate MRSA isolates collected from February 2012 to May 2013 in a tertiary hospital in Suzhou, Eastern China, to explore the molecular epidemiology. All isolates were characterized by *spa* typing, SCC*mec* typing, and detection of genes encoding Panton-Valentine leukocidin (PVL) and toxic shock syndrome toxin (TSST-1). Representative genotypes were also subjected to multilocus sequence typing (MLST). Antibiotic susceptibility testing was performed using BD Phoenix™ Automated Microbiology System. Molecular typing identified 11 clonal complex (CC) and 28 *spa* types, with the CC5-*spa* t002 (29.3%) and CC239-*spa* t037 (14.7%) being the most prevalent. SCC*mec* types II, III, IV, and V were identified in 33.3, 21.3, 23.3, and 21.3% of all isolates, respectively. PVL genes (*lukF/S-PV*) were detected in 11.3% of all isolates and from 6 CCs (5, 8, 59, 88, 239, and 398). The TSST-1 gene (*tst*) was detected in 18.0% of the all isolates, predominantly in CC5 (96.3%). All the *tst-1*-positve CC5 isolates were *spa* t002. Eighteen patients died within 30 days of hospitalization, and the in-hospital 30-day mortality was 12.0%. Multivariable analysis showed that 60 years old (odds ratio [OR] = 7.2, *P* = 0.026), cancer diagnosis (OR = 9.6, *P* = 0.022), and MRSA isolate carriage of *tst-1* (OR = 62.5, *P* < 0.001) were independent factors associated with 30-day mortality. Our study revealed unique MRSA dissemination patterns in our hospital in comparison to those of other regions in China. The finding that *tst-1*-positive CC5 strains were associated with higher mortality highlights the need for strict infection control measures in order to prevent further spread of these strains in our hospital, as well as others.

## Introduction

*Staphylococcus aureus* (*S. aureus*) is one of the most prevalent clinical pathogens isolated from hospital settings, and has recently become widespread in community settings as well. *S. aureus* causes a broad variety of diseases including skin and soft-tissue infections, bacteremia, osteomyelitis, infective endocarditis, and necrotizing pneumonia (Lowy, 1998; Nadig et al., 2010; Tokajian et al., 2010; Song et al., 2013). Following its emergence in the early 1960s, methicillin-resistant *Staphylococcus aureus* (MRSA) has become highly epidemic in many hospitals and health care settings worldwide. Moreover, in recent years distinct community-acquired MRSA (CA-MRSA) strains have also emerged as a cause of invasive and life-threatening infections among young, healthy patients with no significant healthcare exposure (Deleo et al., 2010; Tokajian et al., 2010; Alon et al., 2011; Mediavilla et al., 2012; El-Mahdy et al., 2013).

Molecular typing techniques are routinely used to explore the evolution and epidemiology of MRSA, the most commonly-used being staphylococcal protein A (*spa*) typing (Shopsin et al., 1999; Harmsen et al., 2003), staphylococcal cassette chromosome (SCC) *mec* typing (International Working Group on the Classification of Staphylococcal Cassette Chromosome Elements, 2009), multilocus sequence typing (MLST) (Enright et al., 2000), pulsed-field gel electrophoresis (PFGE) (Tenover et al., 1995), and multiple-locus variable-number tandem repeat analysis (MLVA) (Sabat et al., 2003). Basedon molecular typing, MRSA strains can be divided into various clones, usually denoted by their MLST sequence type (ST) or clonal complex (CC), followed by the SCC*mec* type. Some of these, for example CC8-MRSA-IV (USA300 clone) and CC30-MRSA-IV (Southwest Pacific clone) strains, appear to be pandemic, and have been found on nearly every continent, while others, such as ST59-MRSA-V (Taiwan clone) and ST80-MRSA-IV (European clone), appear to be only regionally disseminated (Mediavilla et al., 2012). In addition, virulence genes such as Panton-Valentine leukocid in (PVL) gene *lukF-PV* and toxic shock syndrome toxin gene *tst-1*, are often associated with certain *S. aureus* clones, and can be used as additional genetic markers with which to characterize MRSA strains. For example, *lukF-PV* has been frequently found in isolates causing community infections, and has traditionally been used as a surrogate maker for CA-MRSA. By contrast, *tst-1* appears to be limited to a handful of clonal lineages, and is most frequently associated with methicillin-susceptible *S. aureus* (MSSA) strains belonging to CC30, while more recently, *tst*-positive CC5 and CC22 MRSA strains have also been documented (Dauwalder et al., 2008; Al Laham et al., 2015).

In China, a multi-drug resistant MRSA clone, defined as ST239 by MLST, is now widely disseminated in different geographic regions, accounting for >75% of all MRSA isolates in two national studies (Liu et al., 2009; Chen et al., 2014). MRSA ST239 strains usually harbor SCC*mec* type III elements, and largely correspond to two *spa* types: t037 and t030. Interestingly, recent studies demonstrated that *spa* t030 has displaced t037 and has thereby become the most frequently-isolated MRSA *spa* type in China (Chen et al., 2010). Second in prevalence following ST239-MRSA-III is the ST5-MRSA-II clone, of which t002 is the most commonly reported *spa* type (19). Other MRSA clones, such as ST398-MRSA-V (live stock-associated) and ST59-MRSA-IV (community-associated), have also been identified (Chen et al., 2010). Previous studies have shown that virulence genes *lukF-PV* and *tst-1* are rarely identified in ST239-MRSA-III and ST5-MRSA-II strains in China. In this study, we report the spread of *tst-1*-harboring ST5-MRSA-II isolates in a tertiary hospital in Suzhou, Eastern China, with multivariate analysis further demonstrating that presence of *tst-1* is an independent risk factor for 30-day mortality.

## Materials and methods

### Bacterial isolates

A total of 150 MRSA isolates collected from February 2012 to May 2013 in a tertiary hospital in Suzhou, China, were included. Identification of *S. aureus* isolates was performed using standard microbiologic methods and the Phoenix System-100 BD Automated Microbiology system (BD Diagnostics, USA). Presence of the *mecA* or *mecC* gene was determined by PCR as described previously (Murakami et al., 1991; Stegger et al., 2012).

### Antimicrobial susceptibility

Isolates of *S. aureus* were inoculated onto the Phoenix panel according to the manufacturer's instructions, following which species identification and antimicrobial susceptibility were determined using the Phoenix System-100 BD Automated Microbiology system (BD Diagnostics, USA). Results of Minimum Inhibitory Concentrations (MICs) were recorded according to Clinical and Laboratory Standards Institute recommendations (CLSI, 2012). *S. aureus* ATCC 29213 was used as a quality control strain for antimicrobial susceptibility testing.

### Molecular typing

All 150 *S. aureus* isolates were characterized by staphylococcal protein A (*spa*) typing (Shopsin et al., 1999), and *spa* types were assigned using eGenomics software (Shopsin et al., 1999; Mathema et al., 2008), with Ridom assignments made using the Spa Server website (http://spa.ridom.de/). In order to avoid confusion, eGenomics *spa* types were shown by the *spa* motif repeats (e.g., *spa* type 2, TJMBMDMGMK), and Ridom *spa* types shown as numbers (e.g., *spa* t030). All MRSA isolates were subjected to SCC*mec* typing using multiplex real-time PCR (Chen et al., 2009). MLST was performed as described previously (Enright et al., 2000) on a representative subset of 30 isolates, with clonal complexes inferred via eBURST analysis (Feil et al., 2004); all other clonal complexes were inferred from *spa* typing data as described previously (Mathema et al., 2008), using both the Ridom Spa Server website and the eGenomics database. Clonal complex sub-groups with distinct genotypic signatures were classified as individual CCs (Mediavilla et al., 2012), e.g., ST239 strains were classified as CC239 rather than CC8. PFGE and MLVA were performed as described previously (Tenover et al., 1995; Sabat et al., 2003).

### Detection of PVL and TSST-1 genes

All isolates were tested for the presence of the genes encoding Panton-Valentine leukocid in (PVL) and toxic shock syndrome toxin (TSST-1). The genes coding for PVL were detected by PCR amplification of *lukS-PV* and *lukF-PV* (Said-Salim et al., 2005), while the gene coding for TSST-1 was detected using a novel real-time PCR assay reported elsewhere (Al Laham et al., 2015).

### Clinical information

For each patient diagnosed with MRSA infection, we recorded demographics, comorbidities, patient location at isolation, specimen source, antimicrobial therapies, vancomycin treatment history, and 30-day mortality. Hospital-onset cases were defined as positive culture occurring ≥3 days after hospitalization. The study was approved by the Institutional Review Boards of the Second Affiliated Hospital of Soochow University.

### Statistical analysis

Characteristics of patients in different groups were compared using Chi-square or Fisher's exact tests for categorical variables, and Wilcoxon rank-sum test for continuous variables. *P* ≤ 0.05 (two-tailed) were considered statistically significant. A multivariable logistic regression model was constructed to identify baseline factors independently associated with 30-day mortality. All variables with *P* ≤ 0.1 in univariate analysis were entered into the multivariable model and a backward stepwise selection process was applied. SPSS, version 22.0 (IBM SPSS, IBM Corporation, Somers, NY) was used for all statistical analyses.

### Ethical approval

The Medical Ethics Committee of Second Affliated Hospital of Soochow University approved this study and all isolates were collected with the patients' written informed consent.

## Results

### Bacterial isolates and patient data

A total of 150 unique MRSA isolates from different specimens, collected from February 2012 to May 2013, were included in this study. Among these, 71 were collected from internal medicine wards (47.3%), 48 from surgical wards (32.0%), and 31 from intensive care unit (ICU) wards (20.7%). All isolates were obtained from inpatients. 60% of patients were more than 60 years old, with an average age of 63, and 74.0% (*n* = 111) are male. A total of 87.3% (*n* = 131) of the cases were hospital-onset. The majority of the isolates were from sputum (*n* = 90, 60.0%), followed by drainage (*n* = 17, 11.3%) and wounds (*n* = 12, 8.0%); 4 isolates were from blood, and 5 were from urine, while the rest of the 22 isolates were from other sites. The Medical Ethics Committee of Second Affiliated Hospital of Soochow University approved this study and all isolates were collected with patient consent in this study.

### Antimicrobial susceptibility

**Figure 1:**
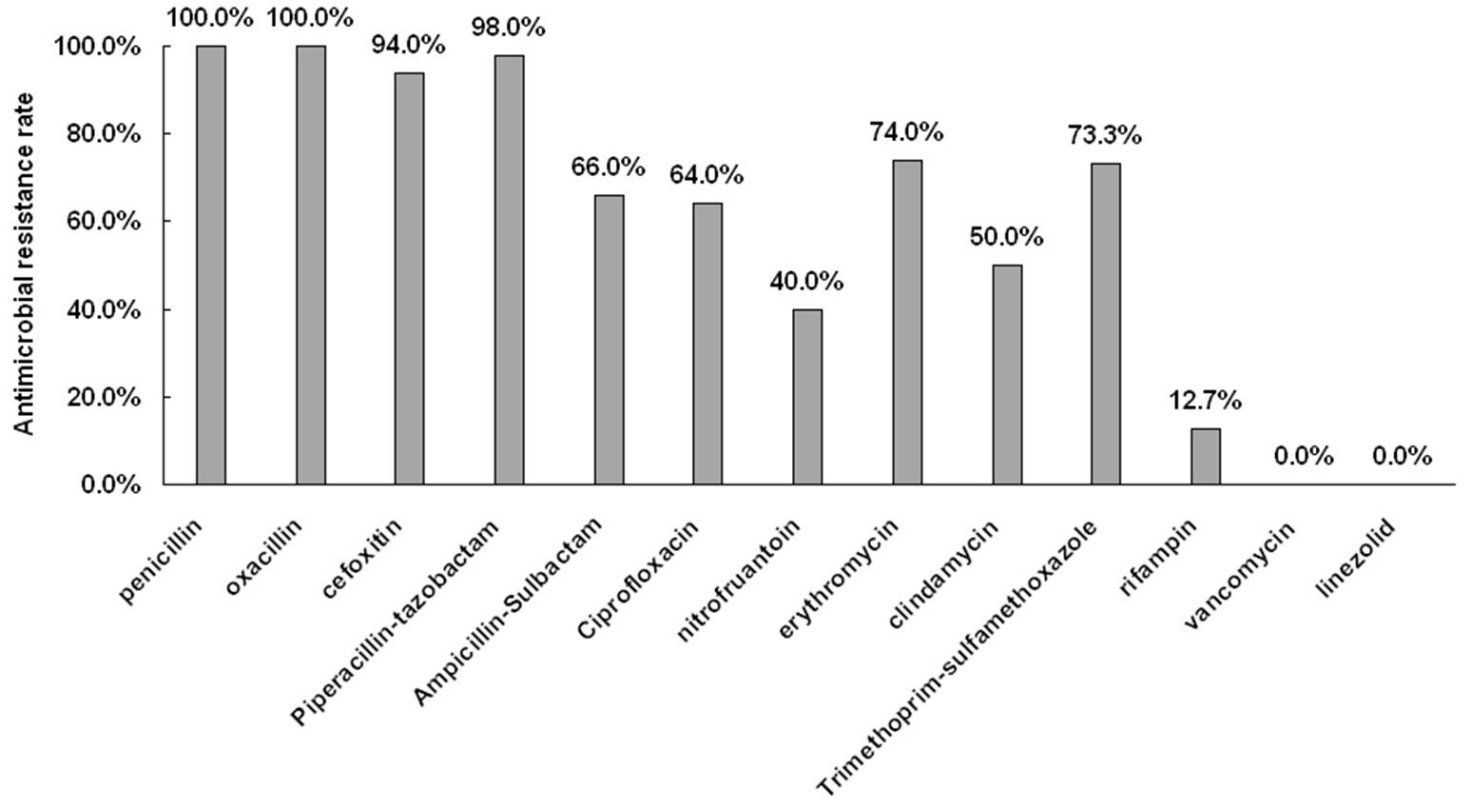
**Antimicrobial resistance rate of 150 MRSA isolates**.

The antimicrobial resistance profiles for all 150 MRSA isolates are shown in Figure 1. All isolates were susceptible to vancomycin and linezolid. The percentage of resistance to both penicillin and oxacillin were 100.0% (150/150), while resistant tocefoxitin, piperacillin-tazobactam, ampicillin-sulbactam, ciprofloxacin, nitrofuantoin, erythromycin, clindamycin, trimethoprim-sulfame thoxazole, and rifampin were 94.0% (141/150), 98.0% (147/150), 66.0% (99/150), 64.0% (96/150), 60.0% (90/150), 74.0% (111/150), 50.0% (75/150), 72.0% (108/150), and 12.7% (19/150), respectively.

### Molecular characteristics of MRSA

Among the 150 MRSA isolates, four SCC*mec* types were identified, including types II (50, 33.3%), III (32, 21.3%), IV (35, 23.3%), and V (32, 21.3%), while1 isolate (0.7%) was non-typeable (NT) (Table 1). A total of 28 *spa* types were identified among the 150 MRSA isolates, belonging to 11 CCs according to eBURST analysis. CC5 isolates were the most common, accounting for nearly one-third (48, 32.0%) of total isolates, followed by CC239 (32, 21.3%), CC59 (19, 12.7%), CC88 (18, 12.0%), CC8 (15, 10.0%), and CC398 (13, 8.7%). One isolate each of CC1, CC7, CC9, CC15, and CC72 was also identified.

**Table 1:** **Molecular characteristics of MRSA isolates**.

**Clonal complex**	***spa* type(Ridom)**	***spa* repeats (eGenomics)**	SCCmec^*^	***lukSF-Pv***	tst-1^*^	**No. of isolates**
CC1	t127	UJFKBPE	NT			1
CC5	t002	TJMBMDMGMK	II		+	25
	t002	TJMBMDMGMK	II	+		4
	t002	TJMBMDMGMK	II			13
	t002	TJMBMDMGMK	IV		+	1
	t002	TJMBMDMGMK	IV			1
	t688	TJMBMK	IV	+		2
	t688	TJMBMK	IV			1
	t2460	TMBBMDMMMK	IV			1
CC7	t091	UJFMBGJAGJ	II			1
CC8	t377	ZAGFMBLO	II			1
	t4223	ZFMBLO	V			1
	t4549	ZBFMFMBLO	V			12
	new	ZBMBLO	III	+		1
CC9	t4132	UKKJAB	V			1
CC15	t085	UJGBBGJAGJ	II			1
CC59	t163	ZDMDMA3KB	IV			1
	t437	ZDMDMOB	II			1
	t437	ZDMDMOB	IV			10
	t437	ZDMDMOB	IV	+		2
	t437	ZDMDMOB	V			2
	t437	ZDMDMOB	V	+		3
	t519	ZDMO	IV			1
CC72	t324	UJGGMDMGGM	IV			1
CC88	t1764	UGFMEEBBBBPE	V			1
	t2310	UGFMEBBBBPE	IV		+	1
	t2592	UGFMEEBBBPE	IV	+		1
	t2592	UGFMEEBBBPE	IV			1
	t3155	UGFMEBBPE	IV			7
	t5348	UFMEEBBBPE	IV	+		1
	t5348	UFMEEBBBPE	IV			2
	t7637	UGFMEEEBBBBPE	II			1
	t8296	UGFMBEBBBPE	II	+		1
	t8296	UGFMBEBBBPE	IV			2
CC239	t030	WGKAQQ	III			5
	t037	WGKAOMQ	III	+		1
	t037	WGKAOMQ	III			21
	t459	WGKAQ	III			2
	t632	XKAQQ	III			2
	unknown	WFFFGKAOMQ	III			1
CC398	t034	XKAOAOBQO	V	+		1
	t034	XKAOAOBQO	V			5
	t571	XKAOAOBO	V			5
	unkown	XAOAOBQ	V			2

* *“+”, positive byPCR. NT, non-typeable*.

The most common *spa* type was t002(29.3%, 44/150), followed by t037(14.7%, 22/150), t437(11.3%, 17/150), t4549(8.0%, 12/150), t3155(4.7%, 7/150), t034(4.0%, 6/150), and t571(3.3%, 5/150), while *spa* t030 was only found in 5 isolates (3.3%). The two predominant genotypes were (a) CC5, *spa* type t002, and SCC*mec* II (*n* = 42, 28.0%), and (b) CC239, *spa* t037, and SCC*mec* III (*n* = 22, 14.7%).

### Prevalence of *pvl* and *tst-1*

Among the 150 MRSA isolates, 11.3% (17/150) tested positive for the presence of the genes coding for PVL (*lukF-PV, lukS-PV*). PVL-positive isolates were identified in six different clonal complexes (Table 1), including CC5, 8, 59, 88, 239, and 398. The two most common *spa* types among PVL-positive isolates were t437 (5/17, CC59) and t002 (4/17, CC5). Among these, 6 (35.3%), 5 (29.4%), and 4 (23.5%) isolates were classified as SCC*mec* type IV, II, and V, respectively.

Similarly, 18.0% of the MRSA isolates (27/150) tested positive for the presence of the TSST-1 gene(*tst-1*), which was detected predominantly in CC5 (*n* = 26, 96.3%); the other *tst-1*-positve isolate belonged to CC88. All the *tst-1*-positve CC5 isolates were *spa* t002. Among the 26 *tst-1*-positive CC5-t002 isolates, 25 were SCC*mec* typeII and 1 isolate was type IV. PFGE and MLVA both showed that these *tst*-positive CC5-t002 isolates have indistinguishable pulsotypes and MLVA patterns (data not shown), suggesting clonal spread.

### Outcome and risk factors

In this study, 18 patients died within 30 days of hospitalization, and the in-hospital 30-day mortality was 12.0%. We then compared the outcomes, clinical characteristics, and MRSA isolate genotypes within three major CCs (CC5, CC239, and CC59) (Table 2). In comparison to cases infected with CC239 or CC59 strains, patients with CC5 infections had significantly higher 30-day mortality (*P* < 0.05). In addition, CC5 strains had a higher frequency of SCC*mec* type II and *tst-1*(*P* < 0.01), and were more likely to be isolated from patients admitted to an ICU (*P* < 0.05) (Table 2).

**Table 2:** **Clinical characteristics and MRSA genotypes of cases with CC5, CC239 and CC59 infections**.

	**CC5**	**CC239**	**CC59**
Number of isolates	48	33	19
**DEMOGRAPHICS**			
Age	65 (59–70)	63 (56–70)	50 (39–61)^*^
Male gender	38 (79.2)	24 (72.7)	16 (84.2)
**UNDERLYING DISEASE**			
Cerebrovascular disease	10 (20.8)	3 (9.1)	5 (26.3)
Cancer	6 (12.5)	4 (12.1)	0 (0.0)
Respiratory infections	13 (27.1)	8 (24.2)	5 (26.3)
Blood stream infections	2 (4.2)	1 (3.0)	1 (5.3)
Degenerative diseases	7 (14.6)	3 (9.1)	1 (5.3)
Burns	0 (0.0)	2 (6.1)	1 (5.3)
Urinary Tract Infections	1 (2.1)	0 (0.0)	1 (5.3)
ICU admission	18 (37.5)	5 (15.2)^*^	2 (10.5)^*^
Hospital onset	45 (93.8)	30 (90.9)	16 (84.2)
**SPECIMEN**			
Sputum	30 (62.5)	21 (63.6)	12 (63.2)
Drainage	5 (10.4)	3 (9.1)	1 (5.3)
Wound	0 (0.0)	2 (10.5)^*^	5 (15.2)
**ANTIMICROBIAL RESISTANCE**			
Penicillin	48 (100.0)	33 (100.0)	19 (100.0)
Vancomycin	0 (0.0)	0 (0.0)	0 (0.0)
Linezolid	0 (0.0)	0 (0.0)	0 (0.0)
Erythromycin	45 (93.8)	32 (97.0)	10 (52.6)^*^
Clindamycin	27 (56.3)	25 (75.8)	8 (42.1)
TMP-SMX	44 (91.7)	27 (81.8)	10 (52.6)
Rifampin	34 (70.8)	15 (45.5)	10 (52.6)
Cefoxitin	47 (97.9)	33 (100.0)	16 (84.2)
Nitrofuantoin	21 (43.8)	20 (60.6)	5 (26.3)
Ciprofloxacin	47 (97.9)	25 (75.8)	18 (94.7)
**STRAIN GENOTYPING**			
**SCC*****mec*** **type**			
SCC*mec* II	42 (87.5)	0 (0.0)^*^	1 (5.3)^*^
SCC*mec* III	0 (0.0)	33 (100.0)^*^	0 (0.0)
SCC*mec* IV	6 (12.5)	0 (0.0)	13 (68.4)^*^
SCC*mec* V	0 (0.0)	0 (0.0)	5 (26.3)^*^
PVL-positive	6 (12.5)	2 (6.1)	5 (26.3)
*tst-1*-positive	26 (54.2)	0 (0.0)^*^	0 (0.0)^*^
Outcome (death)	14 (29.2)	3 (9.1)^*^	1 (5.3)^*^

* *P < 0.05 in comparison to CC5 group. Values are expressed in N (%) or mean (95% confidence interval)*.

We then compared the clinical and molecular characteristics between patients with different clinical outcomes (survival vs. death) (Table 3). The following factors were associated with 30-day mortality in univariate analysis: age, cancer, erythromycin resistance, belonging to CC5, harboring SCC*mec* II, *spa* t002, and presence of *tst-1* (*P* < 0.05). In addition, ICU admission displayed border line significance (*P* = 0.06). Notably, observed 30-day mortality in patients with *tst-1*-positive MRSA was significantly higher than that inpatient with *tst-1*-negative MRSA (51.9 vs. 3.3%, *P* < 0.001). By contrast, no significant difference in 30-day mortality was observed between PVL-positive *vs*.-negative groups (0.0 vs. 13.5%, *P* = 0.13). In multivariable analysis, factors independently associated with 30-day mortality included age greater than 60 years old (odds ratio [OR] = 7.2, 95% confidence interval [95% CI] = 1.26–41.6, *P* = 0.026), cancer diagnosis (OR = 9.6, 95% CI = 1.4–65.7, *P* = *0.02*), and MRSA isolate carriage of *tst-1* (OR = 62.5, 95% CI = 12.0–325.2, *P* < 0.001).

**Table 3:** **Clinical characteristics and outcomes of cases with MRSA infections**.

**Characteristics**	**Survival (*n* = 132)**	**Death (*n* = 18)**	***P***
**DEMOGRAPHICS**			
Age	60 (57–64)	76 (68–83)	**0.003**
Male gender	98 (74.2)	13 (72.2	1.00
**UNDERLYING DISEASE**			
Cerebrovascular disease	25 (18.9)	4 (22.2)	0.47
Cancer	11 (8.3)	5 (27.8)	**0.01**
Respiratory infections	30 (22.7)	7 (38.9)	0.15
Blood stream infections	6 (4.5)	0 (0.0)	0.63
Degenerative diseases	10 (7.6)	2 (11.1)	0.64
Burns	5 (3.8)	0 (0.0)	0.63
Urinary Tract Infections	4 (3.0)	0 (0.0)	1.00
ICU admission	24 (18.2)	7 (38.9)	0.06
Hospital onset	115 (87.1)	16 (88.9)	1.00
Vancomycin treatment	31 (23.5)	5 (27.8)	0.77
**ANTIMICROBIAL RESISTANCE**			
Erythromycin	95 (72)	17 (94.4)	**0.044**
Clindamycin	67 (50.8)	12 (66.7)	0.21
TMP-SMX	95 (72)	15 (83.3)	0.40
Rifampin	77 (58.3)	12 (66.7)	0.50
Cefoxitin	124 (93.9)	17 (94.4)	1.00
Nitrofuantoin	57 (43.2)	7 (38.9)	0.73
Ciprofloxacin	115 (87.1)	16 (88.9)	1.00
**STRAIN GENOTYPING**			
**Clonal complex**			
CC5	34 (25.8)	14 (77.8)	**<0.001**
CC239	30 (22.7)	3 (16.7)	0.76
CC398	13 (9.8)	0 (0.0)	0.23
CC59	18 (13.6)	1 (5.6)	0.47
CC88	18 (13.6)	0 (0.0)	0.13
**SCC*****mec*** **TYPE**			
SCC*mec* II	37 (28.0)	13 (72.2)	**<0.001**
SCC*mec* III	29 (22.0)	3 (16.7)	0.77
SCC*mec* IV	34 (25.8)	1 (5.6)	0.07
SCC*mec* V	31 (23.5)	1 (5.6)	0.12
***Spa*** **TYPE**			
t002	30 (22.7)	14 (77.8)	**<0.001**
t037	20 (15.2)	2 (11.1)	0.75
t437	16 (12.1)	1 (5.6)	0.49
PVL-positive	17 (12.9)	0 (0.0)	0.13
*tst-1*-positive	13 (9.8)	14 (77.8)	**<0.001**

*Values are expressed in N (%) or mean (95% confidence interval). Significant P-values are shown in boldface*.

## Discussion

MRSA is a major nosocomial pathogen worldwide. Infection due to MRSA imposes a high and increasing burden on health care resources, as well as increasing morbidity and mortality. Several major MRSA clones are spreading globally, and they often harbor virulence factors including PVL and TSST-1. Here we characterized 150 non-duplicated MRSA clinical isolates collected from a tertiary hospital in Suzhou, Eastern China. The overall 30-day mortality was as high as 12%. Risk factors for mortality were examined and we found that presence of *tst-1*, age greater than 60 years, and underlying cancer co-morbidity were independent risk factors associated with mortality.

This study revealed some interesting findings. Firstly, CC5 isolates were the main CC in our study, accounting for nearly one third (48, 32.0%) of the total isolates, with the major genotype identified consisting of CC5, *spa* type t002, and SCC*mec* type II (42/150, 28.0%). By contrast, several studies have demonstrated that CC239 is the predominant CC in China, associated with SCC*mec* type III and mainly comprised of *spa* types t037 and t030. A large survey screening a total of 702 MRSA isolates from 18 teaching hospitals in 14 Chinese cities between 2005 and 2006 showed that that the CC239 *spa* type t030 comprised 52.0% of the total isolates, whilet037 accounted for 25.5% (Liu et al., 2009). In addition, the distribution of *spa* types varied among different regions, with t002 (CC5) the most common in northern cities, t037 (CC239) predominant in eastern cities (e.g., Shanghai), and t030 (CC239) the most common in other cities (Liu et al., 2009). Another investigation highlighted a clonal shift from *spa* t037 (from 1994 to 2000) to *spa* t030 (since2000) as the major clone in a Beijing hospital. Similarly, a recent study involving seven hospitals in China showed an increasing prevalence of *spa* type t030, with 80.1% of all MRSA isolates belonging to t030 (Chen et al., 2014). The above studies suggest that ST239 *spa* t030 has replaced t037, which represents the ancestral CC239 *spa* type, as the most frequent MRSA *spa* type in China (Liu et al., 2009; Chen et al., 2010, 2014). By contrast, CC5 *spa* t002 emerged initially in 2002, but has subsequently exhibited a low prevalence rate (Chen et al., 2010). However, our study suggests that this may not be the case in all institutions.

The current study was conducted in Suzhou, which is the largest city near Shanghai, located ~60 miles away. Interestingly, our study showed that t002 was the most common *spa* type in our hospital, whereas over 70% isolates in Shanghai were typed as t037 (Liu et al., 2009). Although located in a similar geographic region, the epidemic pattern of CC and *spa* types in our city was different from that of Shanghai. In addition, our results showed that t037 (16.1%) was more common than t030 (3.6%), which appears to be inconsistent with the pattern of replacement of *spa* t037 by t030 in other studies (Liu et al., 2009; Chen et al., 2010, 2014). Our results therefore suggest differences in geographic distribution of MRSA clones within Chinese hospitals. Specifically, the predominance of CC5 isolates in our hospital is largely due to the high frequency of *tst-1*-positive CC5-MRSA-II, *spa* t002 strains (Table 1).

The CC5 strains in this study have been largely associated with *tst-1*, a gene encoding the toxic shock syndrome toxin TSST-1. In this study, we found that 18.0% (*n* = 27) of all MRSA isolates were *tst-1*-positive, with all but one belonging to CC5. However, *tst-1* is rarely identified in CC5 MRSA strains in China. In a previous multicenter study from China, *tst-1* was detected in 31.4% of isolates tested (including both MSSA and MRSA), but mostly in CC398, CC15, and CC188 (He et al., 2013). Another study from a city in central China has found *tst-1* is mostly associated with CC398, CC59, and CC8, but not CC5 (Liu et al., 2015). Moreover, a recent study from another hospital in Suzhou, the same city as in our study, also reported CC5 as the predominant MRSA genotype, accounting for 50% of all MRSA isolates; however, none of the strains possessed *tst-1* (Li et al., 2015). It appears therefore that *tst-1*-harboring CC5 strains are an emerging clone in our hospital. Alarmingly, our study showed that *tst-1* is an independent risk factor associated with 30-day mortality. The mortality of patients infected by *tst-1*-positive MRSA strains was much greater than that of patients infected by *tst*-negative strains (51.8 vs. 3.5%, *P* < 0.01). Our findings therefore suggest that, in our hospital, *tst-1*-positive CC5-MRSA-II- isolates may be more virulent. Conversely, the presence of another virulence factor, PVL, was not associated with increased mortality. In this study, none of the 17 patients infected by PVL-positive strains died. PVL is commonly found in CA-MRSA strains, and has been associated with severe hospital-acquired pneumonia with increased mortality (Vandenesch et al., 2003; Zhang et al., 2016). However, our results are similar to those of other recent studies of MRSA which did not observe an association between the presence of PVL genes and increased mortality (Peyrani et al., 2011; Haque et al., 2012; Tadros et al., 2013).

Our study had several limitations. Firstly, this study is limited due to the relatively small sample size death cases, which is reflected by the wide range of some confidence intervals in multivariable analysis. Secondly, most of the isolates (60%) were collected from sputum, which is over-represented in the current study. Thirdly, we only tested for the presence of two virulence factors (PVL and TSST-1) while evaluating their associations with outcomes. Therefore, it is possible that other virulence factors co-harbored along with *tst-1* may contribute to the increased mortality observed in the current study. Lastly, this study lacks detailed information about the underlying cause of death, partially due to the nature of retrospective data collection, therefore we were not able to determine the disease specific mortality but total mortality. Nevertheless, our study revealed unique MRSA dissemination patterns in our hospital. The finding that *tst-1*-positive CC5 strains were associated with higher total mortality highlights the need for strict infection control measures in order to prevent further spread of these strains in our hospital, as well as others. Further genome-based analysis of *tst-1*-positive CC5 strains should also be performed to identify additional virulence factors contributing to the high mortality observed in this study.

## Author contributions

MW, work, data analysis and manuscript preparation; YZ, work; JM, manuscript preparation; LC, study design, data analysis and manuscript preparation; BK, manuscript preparation; YS, manuscript preparation; RY, manuscript preparation; HD, study design, work, data analysis and manuscript preparation.

## Funding

This study was supported by the National Natural Science Foundation of China (81572032, 81401636), Six talent peaks project in Jiangsu Province (2016-WSN-112), Gusu key health talent of Suzhou, the Natural Science Foundation for Colleges and Universities in Jiangsu Province (16KJB320006), the Science and Technology Program of Suzhou (SS201638), Pre-research Foundation of Young Workers (SDFEYQN1612), Jiangsu youth medical talents program (QN-867).

### Conflict of interest statement

The authors declare that the research was conducted in the absence of any commercial or financial relationships that could be construed as a potential conflict of interest.
